# Recent Advances of Graphene-Based Strategies for Arsenic Remediation

**DOI:** 10.3389/fchem.2020.608236

**Published:** 2020-12-14

**Authors:** Claudia Foti, Placido Giuseppe Mineo, Angelo Nicosia, Angela Scala, Giulia Neri, Anna Piperno

**Affiliations:** ^1^Department of Chemical, Biological, Pharmaceutical and Environmental Sciences, University of Messina, Messina, Italy; ^2^Department of Chemical Sciences, University of Catania, Catania, Italy

**Keywords:** arsenic, graphene, potable water, magnetic nanomaterials, nanofiltration membrane, nanoadsorbent, remediation

## Abstract

The decontamination of water containing toxic metals is a challenging problem, and in the last years many efforts have been undertaken to discover efficient, cost-effective, robust, and handy technology for the decontamination of downstream water without endangering human health. According to the World Health Organization (WHO), 180 million people in the world have been exposed to toxic levels of arsenic from potable water. To date, a variety of techniques has been developed to maintain the arsenic concentration in potable water below the limit recommended by WHO (10 μg/L). Recently, a series of technological advancements in water remediation has been obtained from the rapid development of nanotechnology-based strategies that provide a remarkable control over nanoparticle design, allowing the tailoring of their properties toward specific applications. Among the plethora of nanomaterials and nanostructures proposed in the remediation field, graphene-based materials (G), due to their unique physico-chemical properties, surface area, size, shape, ionic mobility, and mechanical flexibility, are proposed for the development of reliable tools for water decontamination treatments. Moreover, an emerging class of 3D carbon materials characterized by the intrinsic properties of G together with new interesting physicochemical properties, such as high porosity, low density, unique electrochemical performance, has been recently proposed for water decontamination. The main design criteria used to develop remediation nanotechnology-based strategies have been reviewed, and special attention has been reserved for the advances of magnetic G and for nanostructures employed in the fabrication of membrane filtration.

## Introduction

Arsenic is a ubiquitous element, present in all environmental compartments as well as in living organisms (Merian et al., 2004). It is a component of the earth's crust, minerals, and soils, and it is used as a wood preservative, a component of fertilizers and pesticides, in the mining, metallurgical, glass-making and semiconductor industries. Arsenic toxicity has become a public health problem and an environmental question. The World Health Organization (WHO) estimated that about 180 million people in 50 countries have been exposed to toxic arsenic levels (at least 10 μg/L in drinking water) (International Agency for Research on Cancer IARC, 2012). Arsenic is included among class I carcinogens (International Agency for Research on Cancer IARC, 2012); its toxicity and bioaccumulation greatly depend on its chemical state and on the metabolic pathways in which it is involved (Costa, 2019). Acute and chronic toxicity mechanisms are well-studied, whereas the mechanisms that underlie arsenic-mediated carcinogenesis, including epigenetic alterations, remain largely unknown (Costa, 2019; Nurchi et al., 2020). Arsenic is a metalloid, with four oxidation states (−3, 0, +3, +5), and it exists in a variety of inorganic and organic forms with different toxicity levels, depending on its speciation.

Speciation in aqueous solutions is mostly controlled by redox potential (Eh) and pH. Potential-pH diagrams (Brookins, 1988) show that arsenic in water exists mainly in trivalent or pentavalent form.

Under oxidizing conditions (high E_h_ values), the As (V) species prevail, and their distribution is related to their pH. In natural pH environments (i.e., 4 < pH < 8), As(V) is present as H_2_As${\text{O}}_{4}^{-}$ and HAs${\text{O}}_{4}^{2-}$(Cassone et al., 2018), and the presence of other metal cations must be considered in the natural waters (Nordstrom et al., 2014; Cardiano et al., 2018; Chillè et al., 2018; Giuffrè et al., 2020). Speciation studies performed in presence of Ca^2+^ and Mg^2+^ highlighted the fact that the distribution of As(V) is strongly influenced by the high concentration of these cations and, in sea water, As (V) is mainly present as CaAs${\text{O}}_{4}^{-}$ (46.8%) and MgHAs${\text{O}}_{4}^{0}$ (31.8%), while in fresh water, the main species are HAs${\text{O}}_{4}^{2-}$ and H_2_As${\text{O}}_{4}^{-}$ (31% each), together with CaHAs${\text{O}}_{4}^{0}$ (25.8%) (Chillè et al., 2018).

Under reducing conditions (low E_h_ values), arsenic mainly exists as As (III) and (Cassone et al., 2018) the oxoanions distribution is associated with the pH; up to pH ≈ 9, As (III) is present as arsenous acid H_3_AsO_3_, whereas its anion H_2_As${\text{O}}_{3}^{-}$ represents the stable species for 9 < pH ≤ 11. As (III) can also interact with different classes of chelators (Cassone et al., 2019, 2020; Chillè et al., 2020a,b).

Recently, the techniques developed for arsenic removal, such as membrane filtration, coagulation, adsorption, ion exchange, have been implemented by nanotechnology-based strategies (Ungureanu et al., 2015; Siddiqui et al., 2019). Here, we discuss and summarize (Table 1) the literature on technological advancements in arsenic remediation using graphene-based materials.

**Table 1:** Graphene-based Nanoadsorbents and Membranes.

**Graphene-based system**	**Features**	**Adsorption capacity or rejection**	**References**
GO	GO as adsorbent	As(III) 288 mg/g^**F**^	Reynosa-Martínez et al., 2020
PEI-GO	GO modified with PEI Solid Phase Extraction	As(III) 125 mg/g^**E**^	Ahmad et al., 2018
GO@SiO_2_ and G@SiO_2_	GO or G and SiO_2_ as chromatographic stationary phases	Not specified	Cheng et al., 2018
M-GO	GO, Fe_3_O_4_ nanocomposite as adsorbent	As(III) 85 mg/g^**E**^ As(V) 38 mg/g^**E**^	Yoon et al., 2016
M-RGO	RGO, Fe_3_O_4_ nanocomposite as adsorbent	As(III) 57 mg/g^**E**^ As(V) 12 mg/g^**E**^	Yoon et al., 2016
β-CDs-GO@Fe_3_O_4_	GO modified with β-CDs, Fe_3_O_4_ nanocomposite as adsorbent	As(III) 100.23 mg/g^**E**^ As(V) 99.51 mg/g^**E**^	Kumar and Jiang, 2017
CMGO	Chitosan-magnetic-graphene oxide, nanocomposite as adsorbent	As(III) 45 mg/g^**E**^	Sherlala et al., 2019
mGO/bead	Alginate, GO, Fe_3_O_4_ nanocomposite as adsorbent	As(V) ~99%^**E**^	Vu et al., 2017
GFeN	GO and Fe/Fe_x_O_y_ core-shell as adsorbent	As(III) 306 mg/g^**L**^ As(V) 431 mg/g^**L**^	Das et al., 2020
Mag-PRGO	Partially reduced GO and Fe_3_O_4_ nanocomposite as adsorbent	As(V) 132 mg/g^**E**^	Bobb et al., 2020
SMG	G, Fe (~5 nm) nanocomposite as adsorbent	As(V) 3.26 mg/g^**L**^	Gollavelli et al., 2013
GNP/Fe-Mg	G nanoplates, Fe-Mg nanocomposite as adsorbent	As(V) 103.9 mg/g^**L**^	La et al., 2017b
GNP/CuFe_2_O_4_	G nanoplates, CuFe_2_O_4_ nanocomposite as adsorbent	As(III) 236.29 mg/g^**L**^ As(V) 172.27 mg/g^**L**^	La et al., 2017a
Fe@CuO&GO	Fe/Cu/GO nanocomposite as adsorbent	As(III) 70.36 mg/g^**L**^ As(V) 62.60 mg/g^**L**^	Wu et al., 2019
Fe-GO-Gd	Gd_2_O_3_, Fe_2_O_3_, GO nanocomposite as adsorbent	As(V) 35.84 mg/g^**E**^	Lingamdinne et al., 2020
MG@PDA@PGMA-AET	G/Fe_3_O_4_, Polydopamine, 2-aminoethanethiol as adsorbent	As(III) 62.7 mg/g^**E**^ As(V) 19.3 mg/g ^**E**^	Wang et al., 2019
M-RGO	Fe_2_O_3_ NPs, persulfate (PS) and RGO as catalyst/adsorbent	Total As 89.8%^**E**^	Wu et al., 2020
MAF-RGO	Mn-Al-Fe and RGO as adsorbent	As(III) 402 mg/g^**F**^ As(V) 339 mg/g^**F**^	Penke et al., 2020
G-CNT-Fe 3D	Engineered G, CNT Fe_3_O_4_	Not specified	Vadahanambi et al., 2013
3D G Fe_3_O_4_/aerogel	Fe_3_O_4_/graphene aerogel as adsorbent	As(V) 40.048 mg/g^**L**^	Ye et al., 2015
MGOH	Graphene hydrogel as adsorbent	As (III) 25.1 mg/g^**L**^ As(V) 74.2 mg/g^**L**^	Liang et al., 2019
FeO_x_-CNs	Engineered carbon nanospheres-iron oxide	As (III) 416 mg/g^**F**^ As(V) 201 mg/g^**F**^	Su et al., 2017b
PSU-GO	Membrane produced by phase inversion, used in direct flow filter	As(V) 82.3%^**E**^	Rezaee et al., 2015
PSU-GO	Membrane produced by phase inversion, used in cross-flow filter	As(V) 99%^**E**^	Shukla et al., 2018
GO-coated TFC-NF	PES-supported membrane produced by GO covalent coating on Polyamide, used in cross-flow filter	As(V) 98%^**E**^	Pal et al., 2018a,b
PAN-GO-γ-Fe_2_O_3_	Membrane produced by electrospinning used as batch adsorption	As(V) 95.72%^**E**^	Tripathy and Hota, 2019
PES-GMF	Produced by phase inversion, used in cross-flow filter	As(V) 28.70 mg/g^**E**^	Shahrin et al., 2019
PGLa-Glu-GO-CNT	Membrane produced by spin-casting	As(V) 92%^**E**^ As(III) 96%^**E**^	Viraka Nellore et al., 2015
PLGO	Membrane produced by filtration over cellulose, used as fix-bed adsorption column	As(III) 99.8%^**E**^	Ahmad et al., 2020
DMSPE	Adsorbent deposited on membrane, used in direct flow filter	As(V) 43.9 mg/g^**L**^	Baranik et al., 2018

F *Freundlich model*;

L *Langmuir model*;

E *Experimental*.

### Graphene-Based Materials Employed for Arsenic Remediation

**Figure 1:**
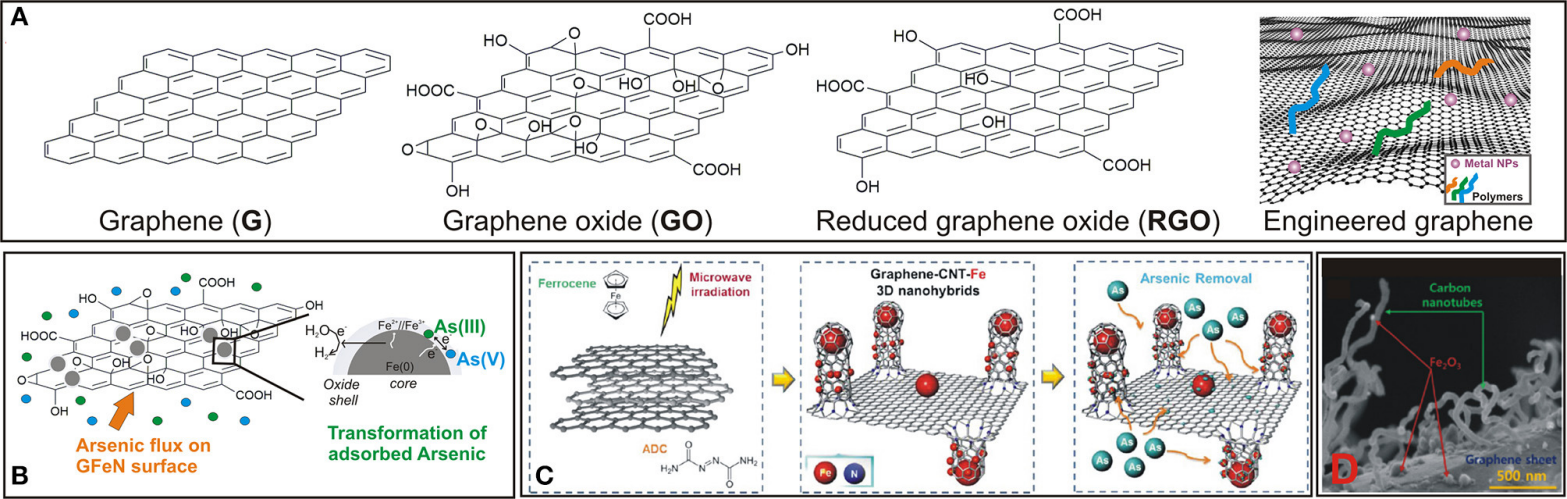
**(A)** Graphene-based materials (graphene, graphene oxide, reduced graphene oxide and engineered graphene). **(B)** Schematic representation of GO-iron nanohybrid (GFeN) and the proposed mechanism involved for arsenic removal. **(C)** Representative example of hierarchically porus 3D assembly (G-CNT-Fe 3D) and the related SEM image **(D)** “Reproduced with permission of (Vadahanambi et al., 2013), (Copyright 2020), American Chemical Society”.

Graphene-based materials (G) include numerous carbon nanomaterials with different morphology, size, shape, chemical surface, and physical-chemical properties (Georgakilas et al., 2012; Yang et al., 2016; Siddiqui and Chaudhry, 2018; Neri et al., 2019; Cordaro et al., 2020; Kokkinos et al., 2020). The G family includes several members such as graphene oxide (GO), reduced graphene oxide (RGO), and their derivatives (e.g., functionalized G and G nanocomposites). Native G showed many remarkable properties, but its poor processability together with the production difficulty on a large scale limited its practical use (Neri et al., 2015b). The development of new derivatives hosting additional functional groups is the main strategy for developing G for practical applications (Neri et al., 2015a). The modification/functionalization processes tune the intrinsic features and allow the assembly of G in various structures (Figure 1).

GO and its composites, in the form of membranes, thin films, paper-like materials, have increasing use in water decontamination, due to their unique physicochemical features (Siddiqui and Chaudhry, 2018).

DFT calculations pointed out that pristine GO binds strongly to heavy metals, like As and Pb, with binding energy of −4.5 and −4.7 eV, respectively, and weakly to Hg (Panigrahi et al., 2018).

The capability of GO to adsorb As species is directly affected by GO oxidation, and its increase from 1.98 to 1.35 (C/O ratio) prompted the As (III) maximum adsorption capacity from 123 to 288 mg/g (Reynosa-Martínez et al., 2020).

The use of GO-based chromatographic stationary phases has allowed the simultaneous separation of different types of arsenic species, avoiding multiple analyses (Reid et al., 2020). Porous GO functionalized with hyperbranched polyethyleneimine (PEI-GO) was proposed for arsenic-selective solid phase extraction (SPE) column. PEI increased the sorption capacity by interacting with both As (III) and As (V) through complexation and electrostatic interactions, respectively (Ahmad et al., 2018).

GO-functionalized silica microspheres (GO@SiO_2_) was investigated for metal speciation analysis of two inorganic arsenicals (arsenite and arsenate) and two organic arsenicals (monomethyl arsenic MMA and dimethylarsenic DMA). No retention by the native GO@SiO_2_ column was observed for the tested arsenicals, that are anions around pH 6.0, as they may be electrostatically expelled. To improve their retaining behaviors, aromatic quaternary ammoniums were added to electrostatically attract arsenic anions. The separation performance of GO@SiO_2_ was compared with that of G@SiO_2_, showing a negligible difference in retention time and resolution, confirming no affinity of oxygenated groups on pristine GO to arsenic anions (Cheng et al., 2018; Zhao et al., 2018).

One of the main concerns related to the use of GO-based materials is the problem of recovery after adsorption, which was resolved using magneto-responsive GO (Hemmati et al., 2018). Iron compounds were reported to form cross-linking with the oxygen functionalities on the surface of carbon materials (Su et al., 2017a). The incorporation of magnetic nanoparticles in GO prevents the aggregation and eases the separation by using an external magnetic field. A comparative study highlighted the fact that As removal was more effective using Fe_3_O_4_-GO composite (M-GO) than Fe_3_O_4_-reduced GO composite (M-RGO), due to the difference in the amount of oxygenated functional groups (Yoon et al., 2016).

Magnetic nanoparticles decorated with β-cyclodextrins-functionalized GO (β-CDs-GO@Fe_3_O_4_ NPs) were proposed as scalable adsorbents of As (III)/As (V) for their excellent water dispersibility and magnetic properties due to the combination of the individual advantages of both materials (Kumar and Jiang, 2017). A nanocomposite based on chitosan and magnetic GO (CMGO) showed the best As (III) adsorption capacity (45 mg/g) at pH 7 (Sherlala et al., 2019).

To increase the water stability, magnetite and GO were encapsulated inside a non-toxic alginate bead (mGO/bead) and the adsorption of Cr (VI) and As (V) from multicomponent systems and contaminated wastewater was evaluated. mGO/bead showed excellent performance (80–100% removal) and recyclability in a complex mixture of heavy metals (Vu et al., 2017).

GO-iron nanohybrid (GFeN) systems were prepared by a sol-gel process for the concurrent removal of As(III)/As(V), without previous oxidation of As(III) to As(V) (Das et al., 2020). As(V) absorption involves electrostatic interactions as well as surface complexation with corrosion products, whereas only surface complexation leads the As(III) absorption. Adsorption capacity was high for both As (V) and As(III) species (Table 1) without iron leaching while it decreased in the presence of ${\text{PO}}_{4}^{2-}$ and ${\text{SiO}}_{3}^{2-}$ ions. GO acts as a reservoir for the electrons released during the oxidation of Fe^0^, allowing the electrons to come back to Fe NPs (Figure 1B).

Magnetite partially reduced GO (Mag-PRGO) nanocomposite obtained *via* laser vaporization-controlled condensation method (Bobb et al., 2020) was exploited to remove As(V). Mag-PRGO showed the ability to remove 100% of As(V) up to 100 ppm final concentration (pH range 4–6), without the loss of iron ions in solution.

GO and ferrocene were used for the preparation of smart magnetic graphene (SMG) by a solvent-free microwave-induced process (Gollavelli et al., 2013). Upon irradiation, GO became graphene and ferrocene decomposed to metallic Fe core (~5 nm in size). SMG showed a maximum As(V) absorption capacity of 3.26 mg/g (Table 1), starting from an arsenic concentration of 5.0 ppm.

Adsorbent systems containing two or more metals or metal oxides were designed to improve arsenic adsorption performance. Graphene nanoplates (GNPs) supported with Fe-Mg binary oxide (La et al., 2017b) or spinel CuFe_2_O_4_ (La et al., 2017a) showed a significant As(V) adsorption. The better adsorption capacity was reached at low pH values, due to the protonation of OH, which attract As(V) oxyanions, whereas the decrease of net positive charge, at higher pH values, leads to a decrease of As(V) adsorption ability. Both systems showed a relevant selectivity toward arsenic anions compared to other ion species.

Fe@Cu&GO systems fabricated by coprecipitation of CuO and Fe_3_O_4_ on GO surface showed good values of absorption for both As(III)/As(V) (Table 1) with a competitive adsorption of phosphate ions (Wu et al., 2019). As(III) adsorption was independent from pH variation, whereas As(V) adsorption decreases under alkali conditions.

Considering the ability of Gadolinium (Gd) oxonium to form binary compounds with arsenic species and its sizeable magnetic moment, a Fe-GO-Gd system (Lingamdinne et al., 2020) was tested for As(V) adsorption. Both ion exchange surface complexation and electrostatic interactions allowed As(V) removal. The adsorption ability decreased in the presence of competitive ions (${\text{SO}}_{4}^{2-}$, ${\text{PO}}_{4}^{3-}$, and ${\text{CO}}_{3}^{2-}$) and after four adsorption/desorption cycles, probably due to the leak of Fe and Gd ions from the GO surface.

Multifunctional magnetic graphene (MG@PDA@PGMA-AET), prepared by surface-initiated ICAR ATRP, was investigated for simultaneous adsorption and sequential elution of As(III) and As(V) (Wang et al., 2019). As(V) oxyanions were absorbed by electrostatic interactions by protonated functional groups of MG@PDA@PGMA-AET, conversely neutral H_3_AsO_3_ species were absorbed by chelation mechanism with –OH, –SH and –NH_2_ groups. The speciation analysis demonstrated a quantitative and simultaneous adsorption of both arsenic species (Table 1), using MG@PDA@PGMA-AET as column packing material.

The heterogeneous Fenton-like system (M-RGO) was proposed for the degradation of 4-aminophenylarsonic (*p*-ASA) and for the adsorption of arsenic species from wastewater (Wu et al., 2020). Removal rate of 89.8% for total As and 98.8% for *p*-ASA were estimated at neutral pH value.

Mn-Al-Fe RGO based hybrid system (MAF-RGO) was proposed to remove arsenic species by electro-sorption and reduction process (Penke et al., 2020). Relevant maximum sorption values for both As(III)/As(V) (Table 1) were estimated by the Freundlich model. Interestingly, the irradiation of MAF-RGO with white light (> 420 nm) increased two-fold the arsenic loading.

Macro/micro/meso porous structures guarantee an excellent permeation of gas and solution, promoting active interior sites. Moreover, 3D G systems are characterized by major mechanical stability avoiding the aggregation phenomenon typical of graphene layers (He et al., 2020). G-CNT-Fe 3D nanohybrid (Figure 1C), composed of CNTs vertically standing on G surface and Fe_n_O_m_ NPs dispersed on CNT and G surfaces, (Vadahanambi et al., 2013) showed a higher performance to capture As(III) species compared to 2D iron-decorated G system. The high surface-to-volume ratio and the mesoporous morphology facilitated the molecular diffusion and the accessibility of iron oxides, which acted as arsenic interactions sites.

Mesoporous 3D G aerogels (GA) homogenously decorated with Fe_3_O_4_ NPs (Ye et al., 2015) showed a higher maximum adsorptive capacity (Table 1) compared with 2D Fe-G systems and porous Fe_3_O_4_. To keep the chemical structure of GO sheets and avoid the damage of oxygen groups, porous 3D magnetic GO hydrogel (MGOH) was prepared by generation of chemical bubbles mixing GO, Fe_3_O_4_ NPs, and polyacrylamide hydrochloride (PA) at room temperature (Liang et al., 2019). MGOH showed good maximum adsorption capacity values (Table 1) with one of the fastest adsorption speeds, reaching equilibrium within only 2 min for both As(III)/As(V) species.

Arsenic capture ability of engineered carbon nanospheres (CNs) with a mesopore/macropore structure depends on the amount of loaded Fe_n_O_m_ (Su et al., 2017b). Fe_n_O_m_ content of 7 and 13 wt% resulted in a maximum adsorption capacity of 246 and 416 mg/g for As(III), and 93 and 201 mg/g for As(V), respectively; at higher Fe_n_O_m_ content a significant decrease in absorption capacity was observed, probably due to the formation of Fe oxide agglomerates that block the pores.

### Graphene-Based Membranes for Arsenic Remediation

Nanofiltration membranes technology is a promising environment-friendly alternative to the conventional adsorbent materials or ion exchange resins (Shukla et al., 2018), providing the rejection of arsenic pollutants by low-cost filtration operations at low transmembrane pressure, through systems suitable to avoid fouling (i.e., cross-flow module) (Sen et al., 2010; Pal et al., 2014). Nanofiltration membranes suitability is mainly affected by the Donnan exclusion principle (Dresner, 1972; Bowen and Mukhtar, 1996; Jye and Ismail, 2017).

The structure and the porosity of polysulfone (PSU)-based membranes including GO can be tuned by exploiting PSU hydrophobicity and GO hydrophilicity (Rezaee et al., 2015; Shukla et al., 2018).

Pure PSU membrane exhibited a sponge-like system with a dense skin layer and a few pores with drop-like ends; the addition of 0.5 (w)% GO resulted in the formation of finger-like pores with closed ends. Further GO loading resulted in a drastic drop of the sponge-like structure, while the pores appeared open-ended and even bigger in size. The negatively charged surface was active in Donnan repulsion of negatively charged pollutants. With an increase in pH, the negative charge of the membrane surface increases, and the predominant arsenate species becomes the divalent ion (${\text{HAsO}}_{4}^{2-}$), enhancing the rejection performances (Rezaee et al., 2015). Although the rejection performance is negatively influenced by the contemporary presence of cations and anions, a higher efficiency of the PSU/carboxylated-GO membrane to reject mixed metal ions solutions than that of pure PPSU was evidenced (Shukla et al., 2018). PSU-based membranes containing GO were prepared also by interfacial polymerization (Pal et al., 2018b). The polyethersulfone (PES) membrane was covered with polyamide, and the residual acid groups belonging to the polyamide-matrix were used to bind a GO layer. This membrane was able to selectively remove ionic As(V) (Table 1), retaining useful metal ions of drinking water, without GO losses in the permeated stream. An economic industrial scale-up was also considered (Pal et al., 2018a).

A PAN-based electrospun composite containing GO and γ-Fe_2_O_3_ was developed by electrospinning of PAN in DMF with GO and γ-Fe_2_O_3_ (Tripathy and Hota, 2019). PAN-GO-γ-Fe_2_O_3_ membrane exhibited high affinity toward As(V) removal (36.1 mg/g) and the presence of anions such as chloride, nitrate, and sulfate do not affect the efficiency, whereas phosphate anions' copresence strongly decreases As(V) chemisorption. As(V) adsorption is proposed as an electrostatic attraction and surface complexation mechanism, operated by the -C-OH and Fe-O groups present on the membrane surface, able to form a complex with arsenate species H_2_As${\text{O}}_{4}^{-}$.

GO-manganese ferrite membranes (PES-GMF, from 0.5 to 2 wt% content) were prepared by dispersing GO-manganese ferrite (GMF) in a polymer mixed matrix of polyvinylpyrrolidone (PVP) and polyethersulfone (PES) that acted as a pore former and support, respectively. GO induced a pore size increase, although high GMF content prompted the agglomerates' formation due to dipole-dipole interactions. GMF increased the membrane hydrophilicity, and the addition of 2 w% of GMF resulted in an increased membrane water flux of 46% in the pure PES membrane. A pH-dependent efficiency was detected: in acidic conditions the electrostatic attraction prevails—positively charged GMF and As(V) in the form of H_2_As${\text{O}}_{4}^{-}$; in alkali conditions the electrostatic rejection occurs (due to the deprotonation of GMF and As(V) in form of HAs${\text{O}}_{4}^{2-}$. The maximum As(V) adsorption capacity of 75.5 mg/g was found for the membrane loaded with 2% GMF (Shahrin et al., 2019).

The adsorptive processes based on electrostatic interactions are suitable only for As(V) species rejection whereas for As(III) removal the affinity of thiolated groups grafted onto engineered membranes was exploited.

A complex 3D porous membrane was synthesized by using GO, single-walled carbon nanotubes (SWCNT) and an antimicrobial PGLa peptide (Viraka Nellore et al., 2015) and tested for the removal of toxic As(III), As(V), Pb(II) and for the disinfection of pathogenic bacteria. Nanofiltration of multiple metal ions solution containing both As(III) and As(V) (10 ppm) and bacteria revealed that 96% of As(III) and 92% of As(V) were rejected from the membrane (Table 1). Although As(III) ions are difficult to remove through nanofiltration, they exhibit high affinity for thiolated groups of glutathione. As a confirmation of the binding affinity of thiolated proteins and As(III), an efficient GO-based membrane suitable for As(III) preconcentration in column phase processes was reported (Ahmad et al., 2020). GO and Bovine Serum albumin (BSA) solution was vacuum-filtered through a cellulose nitrate paper (0.22 μm) to obtain a self-standing polymer-laminated GO membrane (PLGO). BSA was physisorbed onto the GO sheets through electrostatic interactions inducing the formation of interlayer nano capillaries on the membrane surface. PLGO exhibited a maximum adsorption capacity of 140 mg/g, which is about three times higher than that of GO. The presence of other metal ions in solution slightly influences the As(III) selectivity. The recovery and reuse of the membrane do not affect the adsorption efficiency, confirming their usefulness for pre-concentration and speciation of As(III).

A Dispersive Micro-Solid Phase Extraction (DMSPE) membrane was developed by deposition of Al_2_O_3_/GO onto a membrane filter through a vacuum filtration procedure (Baranik et al., 2018). The membrane quantitatively binds As(V) deprotonated species (i.e. H_2_As${\text{O}}_{4}^{-}$ and HAs${\text{O}}_{4}^{2-}$) thanks to the high concentrations of surface hydroxylic groups with a pH-dependent performance.

## Discussion

The selected case studies showed the high potentiality of G nanotechnology to remove As from contaminated water. It is worth noting the ability of engineered graphene to effectively remove complex mixtures of organic and inorganic pollutants from water and its remarkable antimicrobial activity (Karahan et al., 2018).

Although some nanotechnological tools for water purification are already marketed (Khan and Malik, 2019), the use of G in water purification, in particular for As remediation, must be implemented to advance G nanotechnology from lab to the market. Specifically, major concerns such as safety, economic feasibility, and aggregation phenomena, especially in scaled up water purification systems, need to be reasonably addressed. To minimize the health risk, safety issues require a careful evaluation (Caccamo et al., 2020) with the implementation of *in-vivo* studies. The lack of standardized ways for G univocal characterization and the different fabrication methods make the replication of G published results difficult (Piperno et al., 2018). Characterization of G should be carried out by standardized ways to support the new laws for their regulation. REACH (Registration, Evaluation, Authorization, and Restriction of Chemicals) in the European Union is being updated for nanomaterial regulation. Finally, for commercial applications, G would need to be manufactured in standardized way and reduced cost considering that water scarcity is a serious problem in underdeveloped countries.

## Author Contributions

CF, PM, AN, AS, GN, and AP contributed to the design and writing of the mini-review. CF, PM, and AP supervision the work. AP funding acquisition. All authors contributed to the article and approved the submitted version.

## Conflict of Interest

The authors declare that the research was conducted in the absence of any commercial or financial relationships that could be construed as a potential conflict of interest.
